# Inhaled nitric oxide therapy in acute bronchiolitis: A multicenter randomized clinical trial

**DOI:** 10.1038/s41598-020-66433-8

**Published:** 2020-06-15

**Authors:** Aviv Goldbart, Inbal Golan-Tripto, Giora Pillar, Galit Livnat-Levanon, Ori Efrati, Ronen Spiegel, Ronit Lubetzky, Moran Lavie, Lior Carmon, Amit Nahum

**Affiliations:** 1 Saban Pediatric Medical Center, Soroka University Medical Center, Faculty of Health Sciences, Ben-Gurion University, Beer Sheva, Israel; 2 Carmel Medical Center, Haifa, Israel; 3 Sheba Medical Center, Ramat Gan, Israel; 4 Haemek Medical Center, Afula, Israel; 5 Dana-Dwek Children’s Hospital, Tel Aviv Sourasky Medical Center, Sackler School of Medicine, Tel Aviv University, Tel Aviv, Israel

**Keywords:** Respiratory tract diseases, Paediatric research

## Abstract

Currently, there are no approved treatments for infants with acute bronchiolitis, the leading cause for hospitalization of infants worldwide, and thus the recommended approach is supportive. Inhaled Nitric oxide (iNO), possesses anti-viral properties, improves oxygenation, and was shown to be safe in infants with respiratory conditions. Hospitalized infants with acute bronchiolitis were therefore recruited to a prospective double-blinded, multi-center, randomized controlled pilot study. They received intermittent high dose iNO (160 ppm) plus oxygen/air for 30 min or oxygen/air alone (control), five times/day, up to 5 days. Sixty-nine infants were enrolled. No difference was observed in frequencies of subjects with at least one Adverse Event (AE) in iNO (44.1%) vs. control (55.9%); neither was Methemoglobin >7% safety threshold. No drug-related serious AEs (SAEs) were reported. Analysis of Per-Protocol population revealed that length of stay (LOS), time to SpO_2_ ≥92%, and time to mTal clinical score ≤5 improved by 26.7 ± 12.7 (Welch’s t-test p = 0.04), 20.8 ± 8.9 (p = 0.023), and 14.6 ± 9.1 (p = 0.118) hours, respectively, in the iNO group compared to the control. Overall, high dose iNO (160ppm) was safe, well-tolerated, reduced LOS and showed rapid improvement of oxygen saturation, compared to the standard therapy. Further investigation in larger cohorts is warranted to validate these encouraging efficacy outcomes. (Trial registration: NCT03053388)

## Introduction

Bronchiolitis is a viral-induced lower respiratory tract infection and the most common reason for non-elective hospitalization in infants worldwide. In the USA alone, bronchiolitis is responsible for $1.7 billion in hospitalization costs annually with no effective treatment available, beside supportive oxygen therapy and hydration ^1–3^. Moreover, prolonged viral-mediated inflammatory response in bronchiolitis is related to increased risk of long-term respiratory morbidity including asthma and recurrent wheezing ^4–6^. Therefore, there is an unmet need to develop new treatment strategies for infants with bronchiolitis. Inhaled nitric oxide (iNO) has pulmonary vasodilatory properties and has been approved for the treatment of Persistent Pulmonary Hypertension of the Newborn (PPHN) ^7,8^. Endogenous NO production is an essential part of the innate defense mechanism of the human immune system which becomes up-regulated by inducible NO synthase (iNOS) during various inflammatory conditions including microbial and viral infections ^7,8^. Exogenous NO displays broad spectrum anti-microbial and anti-viral activity *in-vitro*, *ex-vivo* and in animal models ^9–16^. Anti-viral activity of NO has been demonstrated *in vitro* against influenza A and B viruses^17^, coronavirus^18^, ectromelia virus^19^, vaccinia virus^19^, and herpes simplex type 1 viruses ^14,19^. A small rescue trial in the treatment of Severe Acute Respiratory Syndrome (SARS) demonstrated NO (<30 ppm) to decrease the spread and intensity of lung infiltrates and improve arterial oxygen saturation^20^. NO also demonstrates protective anti-inflammatory functions ^8,21^, suggesting that NO administration in appropriate concentration could be a highly relevant therapy in bronchiolitis.

*In vitro* studies show that continuous exposure to NO at doses greater than 160 ppm can elicit a strong anti-microbial response ^11,22^. However, continuous exposure to high-dose NO in humans would result in methemoglobinemia, which could lead to decreased oxygen transport and hypoxemia^23^. To overcome this problem, researchers investigated an intermittent iNO regimen (30 min cycles of 160ppm every 3.5 hours) that retained NO’s antimicrobial activity in pre-clinical models ^16,24^ and demonstrated safety and tolerability in healthy adults and cystic fibrosis patients ^25,26^. In a pilot double-blind safety study (n = 43), our group was unable to detect a difference in frequency of AEs and tolerability between intermittent high-dose NO (30 min of 160ppm, 5 times/day) and supportive treatment alone in infants with moderate bronchiolitis. Our previously published results revealed that mean MetHb levels remained well below the 5% safety threshold limit^27^. In this multicenter pilot study, we aimed to determine efficacy, in addition to safety and tolerability, of intermittent high-dose iNO therapy in a larger cohort of hospitalized infants with bronchiolitis.

## Results

**Figure 1:**
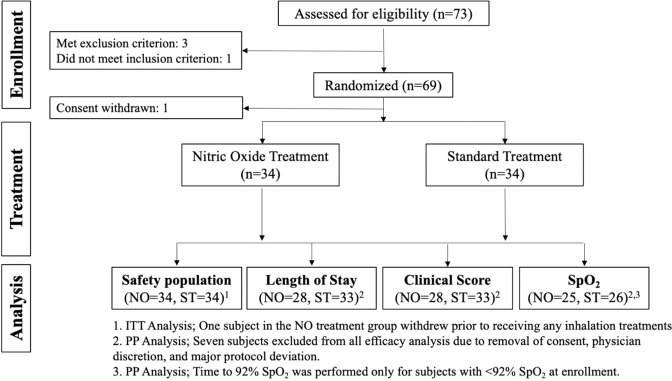
A flow diagram of patient enrollment, exclusion, and outcome analysis. Out of 73 patients that were assessed for the research, we ended with 2 equal groups of 34 pt. that received either iNO or oxygen.

A total of 69 children were recruited out of 73 consecutive, potentially eligible candidates. Among the 69 patients that were recruited, 1 subject was withdrawn (consent withdrawn), without receiving any treatment. Therefore, only 68 subjects participated in the study and were included in the safety analysis (n = 34 per group) (Fig. 1). Baseline characteristics of enrolled patients are summarized in Table 1. No significant differences were found between the baseline characteristics of the two groups; standard treatment and NO + standard treatment.

**Table 1:** Participating patient demographics and baseline data.

Characteristic	Std Treatment (N, Mean ± SD)	NO + Std (N, Mean ± SD)	p value (unpaired t-test)
Gender	21 M, 13 F	21 M, 13 F	—
Age (months)	3.7 ± 2.9	4.0 ± 2.5	0.654
Gestation Week	38.2 ± 1.6	38.1 ± 2.0	0.823
Weight (Kg)	5.7 ± 1.9	5.9 ± 1.7	0.653
Clinical Score (mTal)	8.6 ± 0.9	8.4 ± 1.1	0.422
S_p_O_2_ in room air (%)	88.9 ± 4.1	88.5 ± 3.7	0.679
Body Temp (°C)	37.3 ± 0.9	37.5 ± 0.9	0.370
BP (Sys/Dia)	101.3/59.2	101.7/56.8	—
Heart Rate (beats/min)	153.2 ± 20.9	147.2 ± 22.9	0.270
Respiratory Rate (breaths/min)	58.9 ± 12.7	54.6 ± 8.8	

The Intent-to-treat (ITT) population was defined as all subjects enrolled and randomized into the study who received at least one treatment. There were a total of 68 subjects included in the ITT and evaluated as safety population. Of these, 34 were in the control (Standard Treatment) group and 34 were in the NO treatment (NO + Standard) group. Per-Protocol population consisted of all ITT subjects who completed the study in accordance with the protocol, received at least 4 inhalations and their LOS was at least 20 hours. Figure 1 depicts the numbers of respective patients in the study and the respective Per-Protocol populations that were used for efficacy analysis of LOS, time to clinical mTal score ≤5, and time to sustained SpO_2_ ≥92% are shown. The description of subjects excluded from the efficacy analysis in Per-Protocol population is summarized in Supplementary Table S3.

### Safety

**Figure 2:**
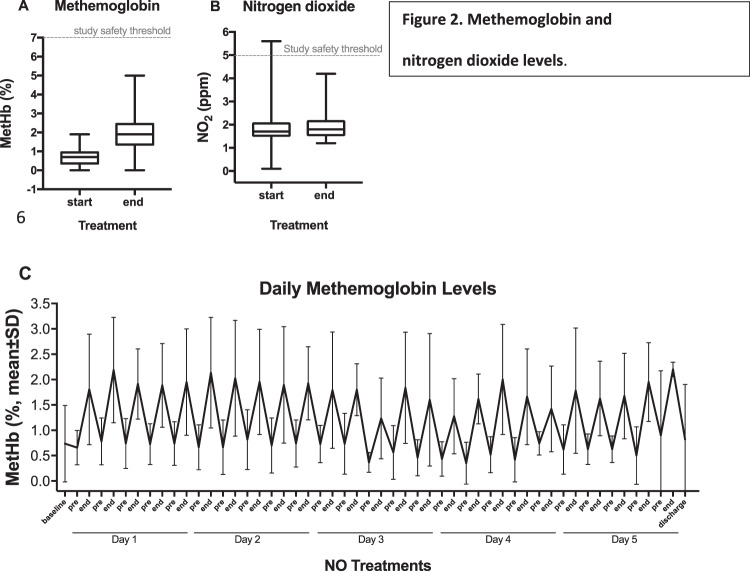
Methemoglobin and nitrogen dioxide levels. Pooled MetHb (panel A) and inspired NO_2_ (panel B) data from the start and end of all treatments (343 total inhalations) are shown in Box-Whisker plots. The dotted lines indicate the study safety threshold limits of 7% for MetHb and 5 ppm for NO_2_. Box-Whisker plot: boxes display 25th to 75th percentile, middle line represents the median, and whiskers extend to minimum and maximum values. (Panel C) shows mean ± SD of daily MetHb levels at baseline, start (pre-) and end (post-) of each NO inhalation, and at discharge.

All MetHb (safety threshold of 7%) and nitrogen dioxide (safety threshold of 5ppm) levels remained within the accepted ranges during all treatments (343 NO inhalations, Fig. 2), with the exception of a single reported NO_2_ levels of 5.6 ppm suspected to be a technical issue since all other values of the same individual were within the lower range. The MetHb levels consistently returned to pre-treatment baseline after each NO inhalation (Fig. 2C). The overall mean MetHb and inspired NO_2_ in the NO + standard treatment group at the end of treatment were found to be 1.89 ± 1.0% and 1.9 ± 0.5 ppm, respectively.

There was no significant difference in the frequency of occurrence of SAEs (Chi-square test, p = 0.525) and AEs (Chi-square test, p = 0.332) between the two treatment groups. A total of 77 AEs were reported in this study with 39 in NO + standard and 38 in standard treatment group. A list of AEs ranked by highest frequency in the NO + standard group is provided in Table 2. The higher frequency of oxygen saturation-decrease in the NO group was attributed to the lower oxygen support during NO inhalations. In order to limit the NO_2_ generation in the delivery circuit during NO inhalation, the maximum delivered oxygen concentration was limited to 40%, whereas the maximum oxygen concentration in the standard support group was 100%. In 13 infants from the NO group an AE of desaturations occurred as compared to only one infant in the control group. Minimal %SpPO2 recorded ranged from 80% to 88% (mean of 85%), and lasted for ≤30 seconds. In 9 cases the specific inhalation was discontinued. A complete list of AEs in both treatment groups is provided in Supplementary Table S4. Vital signs including heart rate, respiratory rate, body temperature, and blood pressure remained comparable between groups and were not affected by the study treatment (Supplementary Table S5).

**Table 2:** Summary of AEs ranked by highest frequency.

*AE preferred term*	Nitric Oxide (n = 34)		Standard Treatment (n = 34)	
	N (subjects)	%	N (subjects)	%
Oxygen saturation-decrease (transient)	15 (9)	38.5%	1 (1)	2.6%
Restlessness	5 (1)	12.8%	1 (1)	2.6%
Pyrexia	3 (3)	7.7%	8 (6)	21.1%
Pneumonia	2 (2)	5.1%	2 (2)	5.4%
Wheezing	2 (2)	5.1%	1 (1)	2.6%
Urinary tract infection	2 (2)	5.1%	—	—
Rash	2 (2)	5.1%	—	—

### Efficacy outcomes

#### Length of Stay (LOS)

**Figure 3:**
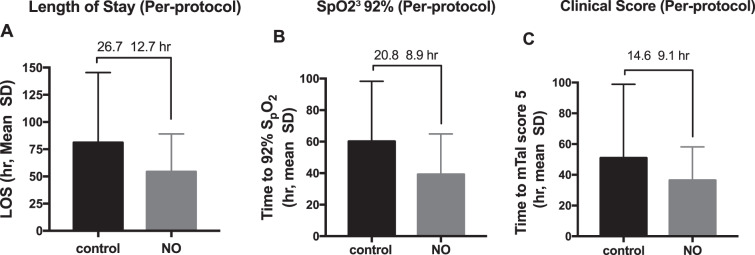
Mean efficacy outcomes show overall improvements in iNO group compared to the control. Mean ± SD for length of stay (LOS, panel A), time to SpO_2_ ≥92% (panel B), and time to clinical score ≤5 (panel C) were analyzed in PP population as described in methods. The value at top of each plot provides the difference between the means.

The primary outcome, LOS (hr), was calculated in Per-Protocol population from the time of enrollment of the subject in the study until the time of the discharge order was written for subjects in the control (n = 33) and std+NO treatment (n = 28) groups (Fig. 3A). The mean LOS was shortened by 26.7 ± 12.7 hours in NO + standard treatment compared to the control group (Welch’s t-test p = 0.04; Age-adjusted, Rank-transformed ANCOVA p = 0.166). The summary statistics of LOS in ITT population is shown in Supplementary Table S6.

#### Time to SpO_2_ ≥92%

The secondary outcome, time to SpO_2_ ≥92% (hr), was calculated in Per-Protocol population from patient enrollment to the time when patient peripheral oxygen saturation stabilized 92% or higher. As shown in Fig. 3B, the NO + standard treatment group (n = 25) revealed a mean of 20.8 ± 8.9 hours reduction in time to SpO_2_ ≥ 92% compared to the control group (n = 26, Welch’s t-test p = 0.023; Age-adjusted, Rank-transformed ANCOVA p = 0.0895). The summary statistics of ITT population is shown in Supplementary Table S7.

#### Time to clinical score ≤5

The time (hours) from patient enrollment to modified clinical Tal score (mTal) of 5 or less was calculated in the Per-Protocol population for the control (n = 33) and NO + standard treatment (n = 28) groups. The mean improvement in this secondary outcome in NO-treated patients was 14.6 ± 9.1 hours compared to the control group (Fig. 3C, Welch’s t-test p = 0.118; Age-adjusted, Rank-transformed ANCOVA p = 0.646). The summary statistics of ITT population is shown in Supplementary Table S8.

Summary statistics of all efficacy endpoints are provided in Table 3.

**Table 3:** Summary statistics of efficacy outcomes, Per-Protocol population.

Summary Statistics	Length of Stay		Time to 92% SpO2		Time to mTal score ≤ 5	
	Std.	NO + Std.	Std.	NO + Std.	Std.	NO + Std.
Number of subjects	33	28	26	25	33	28
Min (hr)	20.3	20.7	14.5	7.5	14.5	7.5
Median (hr)	64.3	46.4	54.1	38.3	42.2	34.7
Max (hr)	283	168	163	106	258	117
Mean (hr)	82.2	55.5	60.8	40.0	51.7	37.1
SD (hr)	63.2	33.6	37.5	24.9	47.2	21.0

## Discussion

This study shows that treating hospitalized infants with bronchiolitis with intermittent high dose iNO is safe, tolerable, and demonstrated trends in improving clinical efficacy endpoints compared to standard treatment, though due to small sample size statistical significance was not reached after adjusting for age as a covariate. The infants who underwent the intervention were discharged one day (26.7 ± 12.7 hours), before their peers in the control group who received only oxygen therapy (in addition to the standard supportive treatment that was shared between the groups), according to current guidelines. They also reached an oxygen saturation of 92%, 20.8 hours faster than the control group. There are several techniques to assess clinical severity of bronchiolitis, and we chose a well-validated clinical score (mTal score) based on subject respiratory rate, accessory muscle use, degree of wheezing, and SpO_2_^28^. Current study findings show that the NO group reached the benchmark of mTal score of 5 or less (score range can be 0–12) within 14.6 ± 9.1 hours faster than controls. These findings are in line with the results of a previous smaller pilot trial completed by Beyond Air Inc., that also compared the safety of intermittent iNO therapy with current standard of care in 43 infants with moderately severe bronchiolitis^27^. The study demonstrated safety, tolerability in NO-treated group and improvement in LOS, time to SpO_2_ of 92%, and mTal score of 5 or less in a subset of patients that were hospitalized more than 24 hours^27^.

For many years, pediatricians are familiar with the use of iNO in the Neonatal (NICU) and Pediatric (PICU) Intensive Care Units for patients with pulmonary hypertension. However, the concept of intermittent high dose NO inhalations for anti-viral/microbial and lung function improvement purposes is new to the pediatric world with promising safety and efficacy outcomes. Broad spectrum antimicrobial activity of endogenous NO as a key part of the host defense system in mammals against bacterial and viral pathogens has long been established ^8,9^. Early pre-clinical studies demonstrated that administration of exogenous NO can inhibit the replication cycle and infectivity of SARS and Influenza A *in vitro* ^11,18^. In a pilot clinical study, Chen *et al*. showed that inhalation of NO can be effective in the treatment of SARS by improving oxygenation and reducing the density of lung infiltrates^20^. In addition, prophylactic iNO therapy was shown to reduce the incidence of bovine respiratory disease, caused by viral/bacterial pathogens, in cattle entering a feedlot^29^. Subsequent clinical studies in adults showed promising safety and tolerability of high-dose intermittent iNO ^25,26^. These observations prompted our group to further investigate the potential of NO therapy in bronchiolitis patients.

The potential side effects of continuous low dose treatment of iNO are well documented. However, not much information regarding intermittent high dose iNO delivery exists. Therefore, this was the primary aim of this study and all subjects were carefully assessed throughout the study and during follow-up period for possible side effects of iNO including those that are well described such as hemoptysis and methemoglobinemia. We found no drug-related SAE and found no significant difference in the frequency of AEs between the iNO and control groups.

Although the concept to use iNO in respiratory tract diseases was “on the wall”, we decided not to introduce this new treatment in severe cases, and thought to first introduce it to moderate severity patients. We aimed to include infants with moderate-severe bronchiolitis (mTal score of 7–10) that are usually admitted to a general pediatric ward. Infants with severe bronchiolitis were admitted to PICU, most of them treated with high flow nasal cannula, CPAP, and rarely with mechanical ventilation. Infants who needed high flow, CPAP or ventilation were excluded from the present study.

One limitation of our study is the set up for the inhalations of the two arms. To any observer it looked totally the same, and it was blinded to the whole staff, except for a technician and one nurse in every shift (for safety reasons) that were un-blinded to the treatment. It was very hard to perform and we believe that this is the closest we could get to double blind in this kind of a study.

Another limitation is the fact we did not rule out that the reason for the improvement in the iNO group was the elevation of their PAP due to pulmonary vasodilation. We could have proved it (though it could be difficult) by performing echocardiography before and after interventions.

Another limitation of this study is not reaching the intended sample size (~94 subjects) due to an unexpectedly shorter bronchiolitis season. For this reason, our study was not powered to reach statistical significance in age-adjusted ANCOVA analysis.

## Conclusion

In conclusion, in this study of hospitalized infants with acute bronchiolitis, the safety and tolerability of inhalations of high dose NO were comparable to those in the standard-supportive treatment. Promising treatment benefits in terms of decreased LOS and time to achieve 92% saturation and accelerated clinical improvement following iNO were presented. Larger scale trials are needed to corroborate the beneficial effect of iNO in bronchiolitis.

## Methods

### Study design

This was a prospective, multi-center, double-blind, randomized study. Seven hospitals in Israel participated in the study during 2017-2018. The study was approved by all medical centers IRB committees and was listed on 15/02/2017 at the NIH site (NCT03053388).

The IRB committees that approved the study in their intuitions include: SOR-Soroka Medical Center, CMC- Carmel Medical Center, EMC- Haemek Medical Center, KMC-Kaplan Medical Center, Central IRB of Clalit Health Services, SMC- Sheba Medical Center, HMO- Hadassah Medical Center, TLV-Sourasky Medical Center. Informed consent was obtained from the legal caregiver of each infant prior to any study-related procedure. Infants were recruited among all pediatric patients admitted with a diagnosis of acute bronchiolitis.

Subjects who met all inclusion criteria and none of the exclusion criteria were enrolled following a detailed explanation of the study protocol to the infant’s caregiver/s. We confirm that all experiments were performed in accordance with relevant guidelines and regulations.

Inclusion criteria included: age up to 12 months of age, born at ≥28 weeks of gestation, acute bronchiolitis requiring in-patient hospitalization expected for 24 hours and more, clinical score (modified Tal score) of between 7 to 10 at screening (without oxygen supplementation). Exclusion criteria: Subjects were excluded if they had alveolar pneumonia, history of previous diagnosis of asthma or life-threatening respiratory distress that requires admission to an intensive care unit for treatment. Subjects with methemoglobin >5% of any cause were also excluded. Detailed inclusion and exclusion criteria are summarized in the Supplementary Tables S1 and S2. A randomization list was generated by statistician prior to the study initiation, using a computerized algorithm, via SAS random number procedure. Following screening procedures, subjects eligible for the study were randomized to receive five 30-minute inhalations of 160 ppm NO combined with O_2_/air (NO treatment) or O_2_/air alone (Standard treatment) every 3-4.5 hours for up to 5 days (maximum 25 inhalations). During inhalations, MetHb, SpO_2_, NO, NO_2_, and FIO_2_ levels were monitored continuously. Respiratory rate was measured prior to and at the end of each inhalation. Additionally, any AE/SAE and concomitant medication was recorded as identified. During hospitalization, several assessments were performed on a daily basis (once or twice daily) until discharge, including abbreviated physical examination, vital signs, SpO_2_ in room air, clinical score, AEs, SAEs, MetHb, concomitant medications, and laboratory tests. The criteria for stopping iNO/placebo (described in p.30 of the attached protocol), were three parameters that included: clinical impression of the attending physician that the patient can be discharged, as well as mTal score of ≤5, and room air SpO_2_ of ≥92%, sustained for two hours. The iNO/placebo was stopped once these three parameters were filled, with no regard to the number of inhalations the patient has already received at that point in time. Subjects were contacted for a follow-up phone call at days 14^+5^ and 30^+5^ from day of admission for additional questioning and safety assessments.

### NO inhalation

Subjects were screened on admission and randomized (1:1) to receive intermittent inhalations of 160 ppm NO along with standard treatment (NO group) or intermittent inhalations of O2/air mixture and standard treatment (control group), for a maximum of 25 inhalations. The treatments were given every 3 to 4.5 hours, for 30 minutes each, using 40% oxygen with NO (NO group) in order to avoid NO_2_ formation, or 100% oxygen (control group).

The study treatment gas was a mixture of NO, air, and oxygen. The NO source was a cylinder of 800 ppm, balanced in nitrogen (Maxima Medical/Gordon Gas) controlled by a needle valve rotameter (CareFusion, G480). The oxygen blend was supplied via air/oxygen blender (Bird, 3800 A) that mixes and controls the oxygen concentration in the enriched air blend also controlled by a needle valve rotameter (Silberman, 4110000). The NO and enriched air gas sources were combined via T-piece prior to delivery to a standard medium concentration pediatric oxygen mask (Hospitak, 223E and Intersurgical INT1190000) for a final NO concentration of 160 ppm (±15 ppm) and a final flow of 5 L/min (±5%). The gas mixture was sampled in close proximity to the patient mask and monitored with an approved NO analyzer (AeroNOx, International Biomed) that provided continuous measurements of NO, NO_2_, and FiO_2_ concentrations.

Supportive treatment was given to all infants. They were all treated with humidified O_2_, FiO_2_ of 100% with a flow of 5 l/min, using oxygen hoods.

### Study blinding

Treatment blindness was maintained by separating the research staff into blinded and un-blinded groups. The un-blinded staff, consisted of nurses, coordinators, and a sub-investigator, administered the inhalations to the infants and monitored MetHb, SpO_2_, FiO_2_, NO, and NO_2_ levels. The blinded group included the primary investigator and all other staff directly involved with patient care. To ensure the integrity of the study, the blinded staff was not present at the room at the time of the treatment. All infants were treated with the same apparatus, hidden at the back of the bed, mimicking the same treatment, so that the parents and most of the ward staff (physicians and nurses), were blinded to the treatment. The technician who actually gave the treatments and followed minute by minute the NO, NO_2_ and MethHb, and one nurse per shift were the only ones that were un-blinded. Separated blinded and un-blinded study documents (i.e., CRF and Source Documents Booklet) were maintained at all times.

### Efficacy and safety variables

In the US, bronchiolitis related to respiratory syncytial virus (RSV) is the leading cause of hospitalization in infants younger than one year, according to American Academy of Pediatrics (AAP). Longer hospitalization presents a high burden on the families and on the hospitals and substantial research and initiatives are focused on the goal to safely reduce bronchiolitis hospitalizations and thereby decrease health care costs. In this study, the primary outcome was LOS assessed from enrollment to the time of physician decision to discharge. The secondary efficacy outcomes were the time required to clinical improvement, measured by modified Tal score (respiratory rate, accessory muscle use, degree of wheezing, and room-air oxygen saturation are assessed)^28^, and time to reach sustained (at least 2 hr) room-air saturation of ≥92% or higher as secondary endpoints^30^. Other secondary outcome was safety and tolerability of iNO, measured by frequency of AEs, methemoglobinemia (7% threshold), and inspired NO_2_ below 5 ppm threshold levels.

### Statistical analyses

Sample size was determined by power analysis based on previous pilot study. The planned sample size was 94 subjects with 1:1 randomization in each treatment group. However, due to unexpectedly shorter bronchiolitis season in Israel, planned sample size was not achieved. The analyses of the primary and secondary endpoints were performed in the Per-Protocol Population from the time of enrollment until the physicians’ decision to discharge. The LOS, time to SpO_2_ ≥92%, and time to mTal score ≤5 were analyzed by Welch’s t-test, as two treatment groups had unequal sample size in per-protocol population (see Table 3), and age-adjusted rank-transformed Analysis of Covariance (ANCOVA). The frequency of AEs between the NO and Standard treatment groups were analyzed by the Chi-square test. All statistical tests were carried out at the 5% (2-sided) significance level.

## Supplementary information

Supplementary Information.

## Data Availability

Deidentified individual participant data will not be made available.
