# Author Correction: Inhaled nitric oxide therapy in acute bronchiolitis: A multicenter randomized clinical trial

**DOI:** 10.1038/s41598-020-70779-4

**Published:** 2020-10-14

**Authors:** Aviv Goldbart, Inbal Golan-Tripto, Giora Pillar, Galit Livnat-Levanon, Ori Efrati, Ronen Spiegel, Ronit Lubetzky, Moran Lavie, Lior Carmon, Abdi Ghaffari, Amit Nahum

**Affiliations:** 1 Saban Pediatric Medical Center, Soroka University Medical Center, Faculty of Health Sciences, Ben-Gurion University, Beer Sheva, Israel; 2 Carmel Medical Center, Haifa, Israel; 3 Sheba Medical Center, Ramat Gan, Israel; 4 Haemek Medical Center, Afula, Israel; 5 Dana-Dwek Children’s Hospital, Tel Aviv Sourasky Medical Center, Sackler School of Medicine, Tel Aviv University, Tel Aviv, Israel; 6 Department of Pathology and Molecular Medicine, Queen’s University, Kingston, ON Canada K7L 3N6

Correction to: *Scientific Reports* 10.1038/s41598-020-66433-8, published online 15 June 2020

Abdi Ghaffari was omitted from the author list in the original version of this Article. This has been corrected in the PDF and HTML versions of the Article, and in the accompanying Supplementary Information file.

The Author Contributions section now reads:

Dr. Aviv Goldbart conceptualized and designed the study, coordinated the investigation between multiple sites, drafted, reviewed, and revised the manuscript. Drs. Inbal Golan-Tripto, Giora Pillar, Galit Livnat-Levanon, Ori Efrati, Ronen Speigel, Ronit Lubetzky, Moran Lavie, Lior Carmon, and Amit Nachum coordinated and supervised the study, data acquisition and interpretation, and critically reviewed the manuscript. Dr. Abdi Ghaffari contributed to the design and writing of the manuscript as well as data analysis of the information presented. All authors approved the final manuscript as submitted and agree to be accountable for all aspects of the work.

