# Supplementary material for: Inhaled nitric oxide therapy in acute bronchiolitis: A multicenter randomized clinical trial
Source: Sci Rep. 2020 Jun 15;10:9605. doi: 10.1038/s41598-020-66433-8 (PMC7295966; doi:10.1038/s41598-020-66433-8)
Supplement: Supplementary file 1 — Supplementary Information. [file 41598_2020_66433_MOESM1_ESM.docx]

**Inhaled nitric oxide therapy in acute bronchiolitis: A multicenter randomized clinical trial**

Aviv Goldbart, MD^1^, Inbal Golan-Tripto, MD^1^, Giora Pillar, MD^2^, Galit Livnat-Levanon, MD^2^, Ori Efrati, MD^3^, Ronen Spiegel, MD^4^, Ronit Lubetzki, MD^5^, Moran Lavie, MD^5^, Lior Carmon, MD^1^, Amit Nahum, MD^1^

**Affiliations:**

1. Saban Pediatric Medical Center, Soroka University Medical Center, Faculty of Health Sciences, Ben-Gurion University - Beer Sheva, Israel
2. Carmel Medical Center – Haifa, Israel
3. Sheba Medical Center - Ramat Gan, Israel
4. Haemek Medical Center – Afula, Israel
5. Dana-Dwek Children’s Hospital, Tel Aviv Sourasky Medical Center, Sackler School of Medicine, Tel Aviv University - Tel Aviv, Israel

**Supplementary Data**

Table S1. Detailed description of study inclusion criteria.

| ***Number*** | ***Inclusion Criteria Description*** |
| --- | --- |
| 1 | Pediatric subjects up to 12-months old.  a. Including subjects born at ≥28 weeks of gestation. |
| 2 | Subjects with acute bronchiolitis requiring in-patient hospitalization expected for 24 hours and more. |
| 3 | Clinical score of between 7 to 10 at screening (evaluated without oxygen supplementation). |
| 4 | Parent/guardian who is willing and able to sign, an informed consent on behalf of the subject. |

Table S2. Detailed description of study exclusion criteria.

| ***Number*** | ***Exclusion Criteria Description*** |
| --- | --- |
| 1 | Subjects diagnosed with alveolar pneumonia on Chest X-ray (including WBC≥ 15,000/ul, and Temp >39°C). |
| 2 | Previous diagnosis of asthma or requirement for asthma medications. |
| 3 | Subjects with >2 previous wheezing episodes. |
| 4 | History of life-threatening respiratory distress that requires admission to an intensive care unit for treatment. |
| 5 | Subjects with history of methemoglobinemia and/or methemoglobin >5% for any cause. |
| 6 | Use of an investigational drug within 30 days before enrolment and/or expected to participate in a new study within 90 days. |
| 7 | History of frequent epistaxis (>1 episode/month) or significant hemoptysis within 30 days prior to enrolment (≥5 mL of blood in one coughing episode or >30 mL of blood in a 24-hour period. |
| 8 | Taken medications such as chronic systemic corticosteroids, CNS stimulants, theophylline or aminophylline, anti-arrhythmic within a certain time period prior to the study. |
| 9 | Unable to comply with the study procedures. |
| 10 | Underlying genetic disorders (including cystic fibrosis) or hypotonia. |
| 11 | Having the following signs or symptoms: 1) known pulmonary (lung) and/or cardiac (heart) congenital malformations 2) an underlying renal, or liver insufficiency, immunodeficiency, encephalopathy; 3) known or suspected foreign body aspiration. |
| 12 | Any reason that, in the opinion of the investigator, may make the subject unfit for this clinical trial. |

Table S3. Description of subjects excluded from efficacy analysis in PP population.

| Analysis | Subject ID | Reason Excluded from Efficacy Analysis |
| --- | --- | --- |
| All Efficacy Analyses | 107 | Subject's parent/legal guardian withdrew consent. |
|  | 633 | Subject’s treatment was discontinued due to Investigator discretion. |
|  | 639 | Subject’s treatment was discontinued due to Investigator discretion. |
|  | 701 | Subject's parent/legal guardian withdrew consent. |
|  | 754 | Subject excluded per sponsor discretion due to inability to comply with treatment. |
|  | 851 | Subject excluded due to major protocol deviation. |
|  | 501^1^ | Subject excluded due to major protocol deviation |
|  | 623 | Subject's parent/legal guardian withdrew consent prior to receiving any inhalation treatments. |
| 92% Saturation | 613 | Subject entered the study with saturation level≥92%. |
|  | 615 | Subject entered the study with saturation level≥92%. |
|  | 625 | Subject entered the study with saturation level≥92%. |
|  | 704 | Subject entered the study with saturation level≥92%. |
|  | 751 | Subject entered the study with saturation level≥92%. |
|  | 752 | Subject entered the study with saturation level≥92%. |
|  | 753 | Subject entered the study with saturation level≥92%. |
|  | 801 | Subject entered the study with saturation level≥92%. |
|  | 951 | Subject entered the study with saturation level≥92%. |
|  | 954 | Subject entered the study with saturation level≥92%. |

Table S4: AE comparison – NO treatment group and Standard Treatment

| **System Organ Class** | **Nitric Oxide Treatment** | | | | **Standard Treatment** | | | |
| --- | --- | --- | --- | --- | --- | --- | --- | --- |
|  | **subjects** | **%** | **events** | **%** | **subjects** | **%** | **events** | **%** |
| **All events** | **15** | **44.1** | **39** | **100** | **19** | **55.9** | **38** | **100** |
| **Investigations** | **10** | **29.4** | **16** | **41.0** | **1** | **2.6** | **1** | **2.6** |
| Oxygen saturation decreased | 9 | 26.5 | 15 | 38.5 | 1 | 2.6 | 1 | 2.6 |
| Enterococcus test positive | 1 | 2.9 | 1 | 2.6 |  |  |  |  |
| **Infections and infestations** | **7** | **20.6** | **7** | **17.9** | **10** | **29.4** | **13** | **34.2** |
| Pneumonia | 2 | 5.9 | 2 | 5.1 | 2 | 5.9 | 2 | 5.3 |
| Urinary tract infection | 2 | 5.9 | 2 | 5.1 |  |  |  |  |
| Respiratory tract infection | 1 | 2.9 | 1 | 2.6 |  |  |  |  |
| Conjunctivitis |  |  |  |  | 2 | 5.9 | 2 | 5.3 |
| Sepsis |  |  |  |  | 1 | 2.9 | 1 | 2.6 |
| Adenovirus infection |  |  |  |  | 1 | 2.9 | 1 | 2.6 |
| Gastroenteritis |  |  |  |  | 1 | 2.9 | 1 | 2.6 |
| Otitis media |  |  |  |  | 4 | 11.8 | 4 | 10.5 |
| **Respiratory, thoracic and mediastinal disorders** | **3** | **8.8** | **3** | **7.7** | **6** | **17.6** | **7** | **18.4** |
| Wheezing | 2 | 5.9 | 2 | 5.1 | 1 | 2.9 | 1 | 2.6 |
| Respiratory distress | 1 | 2.9 | 1 | 2.6 | 1 | 2.9 | 1 | 2.6 |
| Cough |  |  |  |  | 2 | 5.9 | 2 | 5.3 |
| Dyspnoea |  |  |  |  | 2 | 5.9 | 2 | 5.3 |
| Rhinorrhoea |  |  |  |  | 1 | 2.9 | 1 | 2.6 |
| **General disorders and administration site conditions** | **3** | **8.8** | **3** | **7.7** | **7** | **20.6** | **9** | **23.7** |
| Pyrexia | 3 | 8.8 | 3 | 7.7 | 6 | 17.6 | 8 | 21.1 |
| Oedema peripheral |  |  |  |  | 1 | 2.9 | 1 | 2.6 |
| **Skin and subcutaneous tissue disorders** | **2** | **5.9** | **2** | **5.1** |  |  |  |  |
| Rash | 2 | 5.9 | 2 | 5.1 |  |  |  |  |
| **Injury, poisoning and procedural complications** | **2** | **5.9** | **2** | **5.1** | **1** | **2.9** | **1** | **2.6** |
| Exposure via inhalation | 2 | 5.9 | 2 | 5.1 |  |  |  |  |
| **Cardiac disorders** | **1** | **2.9** | **1** | **2.6** | **1** | **2.9** | **1** | **2.6** |
| Bradycardia | 1 | 2.9 | 1 | 2.6 | 1 | 2.9 | 1 | 2.6 |
| **Psychiatric disorders** | **1** | **2.9** | **5** | **12.8** | **1** | **2.9** | **1** | **2.6** |
| Restlessness | 1 | 2.9 | 5 | 12.8 | 1 | 2.9 | 1 | 2.6 |
| **Congenital, familial and genetic disorders** |  |  |  |  | **1** | **2.9** | **2** | **5.3** |
| Patent ductus arteriosus |  |  |  |  | 1 | 2.9 | 1 | 2.6 |
| Atrial septal defect |  |  |  |  | 1 | 2.9 | 1 | 2.6 |
| **Gastrointestinal disorders** |  |  |  |  | **3** | **8.8** | **3** | **7.9** |
| Aphthous ulcer |  |  |  |  | 1 | 2.9 | 1 | 2.6 |
| Umbilical hernia |  |  |  |  | 1 | 2.9 | 1 | 2.6 |
| Diarrhea |  |  |  |  | 1 | 2.9 | 1 | 2.6 |

Table S5. The summary of monitored safety parameters.

| ***Parameter*** | ***Nitric Oxide Treatment*** | | | | | | ***Standard Treatment*** | | | | | |
| --- | --- | --- | --- | --- | --- | --- | --- | --- | --- | --- | --- | --- |
|  | ***N**** | ***Mean*** | ***SD*** | ***Min*** | ***Median*** | ***Max*** | ***N**** | ***Mean*** | ***SD*** | ***Min*** | ***Median*** | ***Max*** |
| *Heart Rate (beat/min), start of treatment* | 328 | 145 | 19.3 | 101 | 144 | 200 | 384 | 145 | 20.7 | 83 | 146 | 203 |
| *Heart Rate (beat/min), end of treatment* | 327 | 144 | 19.8 | 94 | 143 | 218 | 383 | 142 | 20.7 | 78 | 142 | 197 |
| *Respiratory Rate (breath/min), start of treatment* | 342 | 51.8 | 12.7 | 23 | 50 | 99 | 387 | 55.6 | 12.9 | 29 | 55 | 100 |
| *Respiratory Rate (breath/min), end of treatment* | 322 | 51.7 | 11.9 | 22 | 51 | 86 | 375 | 55.6 | 12.9 | 26 | 56 | 100 |
| *Blood Pressure, systolic (mmHg)* | 120 | 98.5 | 11.3 | 67 | 98 | 132 | 142 | 98 | 12.2 | 74 | 98.5 | 126 |
| *Blood Pressure, diastolic (mmHg)* | 120 | 55.8 | 11.7 | 30 | 56 | 89 | 142 | 56.3 | 12.8 | 30 | 55 | 108 |
| *Body Temperature (^o^C)* | 175 | 37.2 | 0.7 | 35.9 | 37.1 | 39.6 | 190 | 37 | 0.7 | 35.6 | 36.9 | 40.2 |

* N represents the total number of measurements for each readout during the study. SD: standard deviation.

Table S6. Summary statistics of LOS (hours), from enrollment to discharge, ITT Population.

| LOS (hours) from enrollment to discharge | NO Treatment | Control |
| --- | --- | --- |
| *N* | 34 | 34 |
| *Mean* | 69.8 | 81.4 |
| *SD* | 56.2 | 62.4 |
| *Min* | 20.7 | 20.3 |
| *Median* | 46.9 | 63.8 |
| *Max* | 270.7 | 282.8 |
| Welch’s t-test (compared to Control) | 0.4246 | |
| Rank-transformed ANCOVA, age adjusted (compared to Control) | 0.4037 | |

Table S7. Summary statistics of time from enrollment to sustained SpO2 ≥ 92%, ITT population.

| Time (hours) to SpO_2_ ≥ 92% | NO Treatment | Control |
| --- | --- | --- |
| *N* | 31 | 27 |
| *Mean* | 61.6 | 65.8 |
| *SD* | 57.6 | 44.7 |
| *Min* | 7.5 | 14.5 |
| *Median* | 40.9 | 61.6 |
| *Max* | 193.7 | 193.7 |
| Welch’s t-test (compared to Control) | 0.7585 | |
| Rank-transformed ANCOVA, age adjusted (compared to Control) | 0.3918 | |

Table S8. Summary statistics of time from enrollment to mTal score≤5, ITT population.

| Time (hours) to mTal score≤5 | NO Treatment | Control |
| --- | --- | --- |
| *N* | 34 | 34 |
| *Mean* | 47.3 | 51.7 |
| *SD* | 39.2 | 46.5 |
| *Min* | 7.5 | 14.5 |
| *Median* | 40.0 | 42.5 |
| *Max* | 192.7 | 258.2 |
| Welch’s t-test (compared to Control) | 0.6751 | |
| Rank-transformed ANCOVA, age adjusted (compared to Control) | 0.9553 | |
